# One-pot synthesis of novel 1*H*-pyrimido[4,5-*c*][1,2]diazepines and pyrazolo[3,4-*d*]pyrimidines

**DOI:** 10.1186/1860-5397-2-5

**Published:** 2006-03-23

**Authors:** Dipak Prajapati, Partha P Baruah, Baikuntha J Gogoi, Jagir S Sandhu

**Affiliations:** 1 Department of Medicinal Chemistry, Regional Research Laboratory, Jorhat 785006, Assam, India; 2 Department of Chemistry, Panjabi University, Patiala 147 002, Punjab, India

## Abstract

Novel 1*H*-pyrimido [4,5-*c*][1,2]diazepines **3 & 4** and pyrazolo [3,4-*d*]pyrimidines **6** were regioselectively synthesised by the reaction of 1,3-dimethyl-6-hydrazinouracils **1** with various α,β-unsaturated compounds **2** and α-ketoalkynes **8** in excellent yields.

## Introduction

The importance of uracil and its annelated substrates is well recognized by synthetic [1–3] as well as biological chemists. [4–6] With the development of clinically useful anticancer and antiviral drugs (AZT, DDI, DDC, BVDU) there has recently been remarkable interest in the synthetic manipulation of uracils. 4-Deazatoxaflavin (1,6-dimethyl-1,5,6,7-tetrahydro pyrimido [4,5-*c*]pyridazine-5,7-dione), inhibits the growth of Pseudomonas 568 and also binds to herring sperm DNA.[7] However, until the emergence of HEPT[8] as a potent and selective inhibitor of HIV-1, no attention was given to the synthetic manipulation at the 6-position of uracils. Also the synthetic exploitation of the nucleophilic double bond of uracil is an undeveloped field in view of a great variety of potential products. [9–12] There have been reports for direct functionalisation of uracil using the C5-C6 double bond via thermolytic[13] and photocycloaddition reactions.[14–15] The heteroannulation of uracils usually require either forcing conditions[16–17] or relatively longer synthetic pathways.[18] Also, pyrazolo [3,4-d]pyrimidines are a class of naturally occurring fused uracils that possess a wide range of biological activity.[19] Allopurinol (6-dehydroxy-pyrazolo [3,4-d]pyrimidine), an effective xanthine oxidase inhibitor,[20] is in clinical use for controlling gout and related metabolic disorders.[21] In continuation of our studies on uracil analogues [22–24] we describe the results of our study on the reaction of 1,3-dimethyl-6-hydrazino uracils **1** with various α,β-unsaturated compounds and α-ketoalkynes, which give access to an efficient unprecedented one-pot synthesis of novel pyrimido [4,5-*c*][1,2]diazepine-6,8-diones **3** or **4** and pyrazolo [3,4-*d*]pyrimidines **6** in excellent yields. A previous synthesis of pyrimido [4,5-*c*][1,2]diazepines reported by Mallory *et al.*[25] involved the reaction of hydrazinoisocytosines and α,γ-diketoesters, in which pyrimido [4,5-*c*]pyridazine was also formed as a side product. However, they have reported[26–27] that the reaction of 6-hydrazinouracil with α-keto acids or symmetrical and unsymmetrical vicinal dicarbonyl compounds gives exclusively the pyrimidopyridazine. A literature survey revealed no other reports on the synthesis of pyrimido[1,2]diazepines. In contrast benzodiazepines are extensively studied[28] biologically active molecules and several of its derivatives are drugs in the market. Since the introduction of Librium as a minor tranquiliser, a large number of seven-membered heterocyclic compounds with the benzodiazepine moiety have been synthesized and tested for psychotropic properties.[29] This moiety has been found to represent a versatile template in peptidomimetic design and it is also found in several other compounds of biological importance including anti-tumor antibiotics and inhibitors of HIV-1 transcriptase.[30] New methods continue to appear in the literature describing the synthesis of novel benzodiazepine analogues[31], and emphasis is placed on the alteration and replacement of benzene ring with a pyrimidine derivative, which will not only increase the synthetic scope of this hitherto under-developed reaction, but it will expand the synthetic versatility of uracil derivatives and provide diversity in the nature of the heterocyclic motif in a targeted library of potential products.

## Results and discussions

The synthesis of pyrimido [4,5-*c*][1,2]diazepine-6,8-dione **3a** was accomplished by reacting equimolar quantities of 1,3-dimethyl-6-hydrazino uracil **1** and α,β-unsaturated carbonyl compound **2a** in ethanol under reflux for 3 h (monitored by TLC). The suspension of 6-hydrazino uracil **1** first disappeared with the addition of enone **2a** to a clear solution. After completion and usual work-up, the product **3a** was obtained in 85% yield. The reaction did not indicate the formation of any six-membered ring product pyrimido [4,5-c]pyridazine **5** as expected via [4+2] cycloadditions, which would have occurred through involvement of an azine (obtained from oxidation of hydrazine group). Similarly other α,β-unsaturated carbonyl compounds **2b-e** were reacted and the corresponding pyrimido[1,2]diazepines **3b-e** were obtained in 78–90% yields and were characterized fully by spectroscopic and elemental analyses. The IR (KBr) band at 3389 cm^-1^ (NH, stretch) and 1522 cm^-1^ (NH, bending) revealed that the compound has an NH group. Other absorption bands at 1697 and 1607 cm^-1^ indicated the presence of cyclic imide system. In the mass spectrum the strong molecular ion peak MS *m/z* at 390 (M^+^) suggested that the products were formed by water loss from the two substrates. The ^13^C NMR showed that the product contained one aliphatic CH_2_ and one aliphatic CH besides other expected moieties. No C=O group (other than the cyclic imide system) was indicated either from the IR or ^13^C NMR spectrum. Interestingly, when mesityl oxide was reacted with the 1,3-dimethyl-6-hydrazinouracil **1** under the identical condition, we did not observe the formation of pyrimido [4,5-*c*][1,2]diazepine of the type **3** as we obtained with other enones. Here, we obtained the corresponding pyrimido [4,5-*c*][1,2]diazepine **4**, a tautomeric form of **3** in 90% yield. The structure **4** was fully characterized through spectroscopic data and elemental analysis. The IR (KBr) band at 1589 cm^-1^ reveals the presence of (C=N), which confirmed involvement of hydrazine group in the reaction. The other absorption bands at 1718 and 1676 cm^-1^ indicated the presence of cyclic imide system. Thus no C=O group (other than the cyclic imide system) was indicated either from IR or ^13^C NMR spectrum. The ^1^H NMR spectra showed the absence of C-5 proton of the uracil moiety which supported the involvement of a cyclisation process. The absence of NH/OH group could be explained either from IR absorption or ^1^H NMR spectra because the compound **4** has no other D_2_O exchangeable signal. In the mass spectrum the strong molecular ion peak MS *m/z* at 250 (M^+^) suggested that the products were formed by water loss from the two substrates. It is also notable here that the pyrimidodiazepines synthesised by Mallory *et al* is susceptible to ring-opening/ring-closure rearrangement reactions. Depending on the nature of the substituents, their location, and reaction conditions, the pyrimidodiazepines were converted into either pyrido [2,3-*d*]pyrimidines, pyrazolo [3,4-*d*]pyrimidines or a pyrimido [4,5-*c*]pyridazine.[14] But on heating pyrimidodiazepine **3a** with 1 N HCl above 90°C for about 2 h, we observed the formation of corresponding pyrazolo [3,4-d]pyrimidines in poor yields (15%). To enhance the yield further, increase of reaction time also did not give any fruitful results rather decomposition of starting material occurred.

**Scheme 1:**
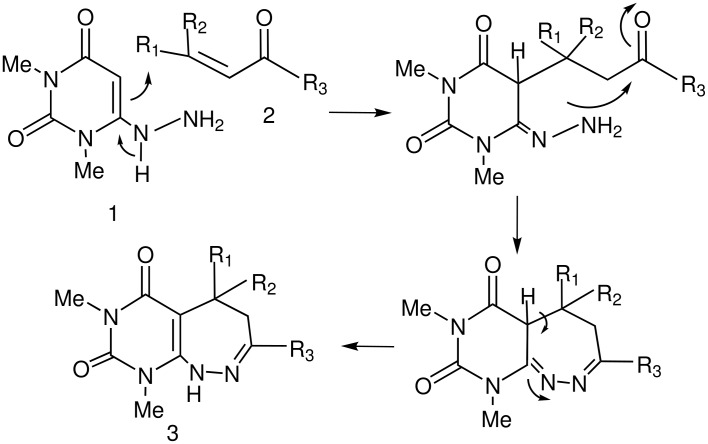
Mechanism for the formation of Pyrimido [4,4-c][1,2]diazepines.

Although we could not isolate any intermediate from the reaction mixture a plausible mechanism for the formation of the products entails an initial Michael reaction of **1** to **2** followed by cyclodehydration possibly by a Bohlmann-Ratz type[32] reaction leading to a seven-membered ring products **3** (Scheme 1). This mechanism explains the formation of both the products **3** &**4** i.e. the presence of an aliphatic CH and loss of PhCN from M^+^, although they differ in their tautomeric forms. Further study of this effective synthetic method is in progress.

**Scheme 2:**
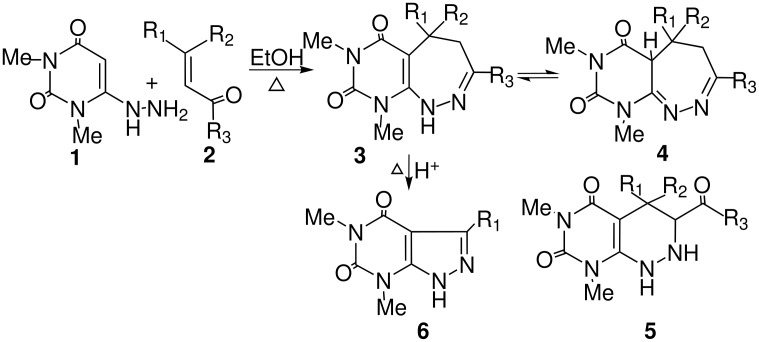
Reagents and conditions: i) EtOH, reflux.

**Scheme 3:**
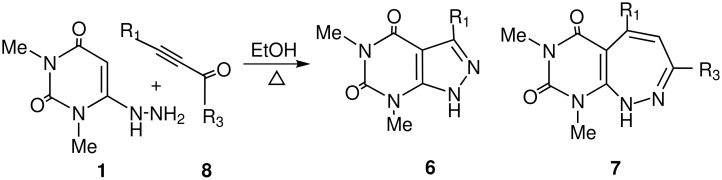
Reagents and conditions: i) EtOH, reflux.

**Scheme 4:**
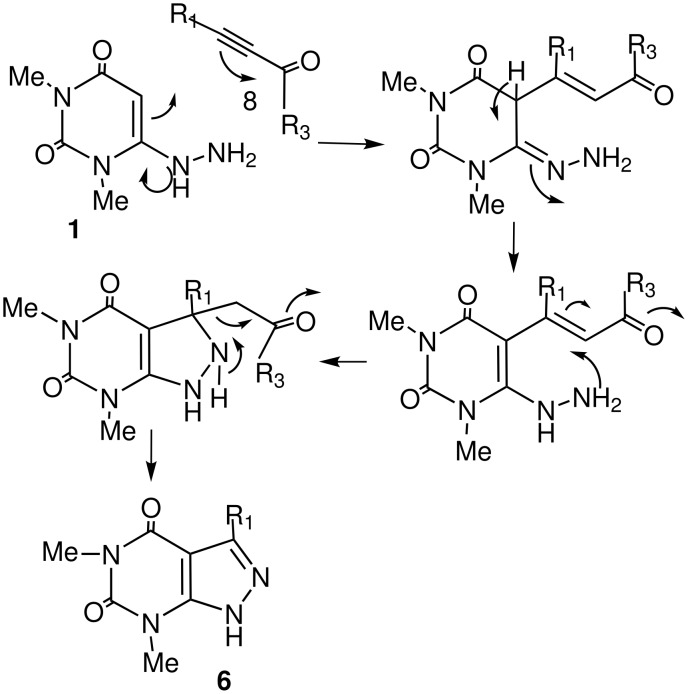
Mechanism for the formation of Pyrazolo [3,4-d]pyrimidines.

To investigate further the synthetic scope this reaction, we reacted 1,3-dimethyl-6-hydrazinouracil **1** with an α-ketoalkyne (3-hexyn-2-one) in refluxing ethanol, the reaction proceeded somewhat differently from that with an enone (Scheme 2), as the intermediate could undergo a 5-*exo-trig* cyclisation in preference to 7-*exo-trig*. Here, we have isolated the corresponding 2,4-dioxo-pyrazolo [3,4-*d*]pyrimidine **6a** in 80% yield instead of the expected seven-membered pyrimido[1,2]diazepine **7** or any other product (Scheme 3). The structure of the product **6a** thus obtained was confirmed unambiguously by high resolution spectral techniques. To examine further the scope of the heteroannulation reaction, 4-phenyl-3-butyn-2-one was treated with 1,3-dimethyl-6-hydrazinouracil **1** in refluxing ethanol for 4 h and the corresponding pyrazolo [3,4-*d*]pyrimidine derivative was isolated in 78% yield. It is notable that pyrimido[1,2]diazepine on heating in hot acid produces pyrazolo [3,4-*d*]pyrimidines, most likely by hydrolytic ring opening at the 'hydrazone' bond followed by a retro-aldol reaction and ring closure.[25,33–34] In contrast, we synthesised the corresponding pyrazolo [3,4-*d*]pyrimidines in high yields under mild conditions without heating in any strong acid. Here the reaction proceeded initially by the Michael addition of α-ketoalkyne **8** to **1** followed by cyclisation to a 5-membered ring (Scheme 4).

**Table 1:** Characteristics for Pyrimido [4,5-*c*][1,2]diazepines **3** &**4** and pyrazolo [3,4-*d*]pyrimidines **6**.

Products	R^1^	R^2^	R^3^	Reaction Time, h	Yield^a^ %	M.p. °C

**3a**	C_6_H_4_OMe-*p*	H	Ph	3	85	198–200
**3b**	Ph	H	Ph	3	78	225–226
**3c**	Ph	H	Me	5	80	158–160
**3d**	C_6_H_4_-Cl-*p*	H	Me	5	80	192–193
**3e**	H	H	Me	3	90	190–191
**4**	Me	Me	Me	3	90	156–157
**6a**	Et	-	-	2	80	237–239
**6b**	Ph	-	-	4	78	242–244
**6c**	Et	-	-	4	75	237–239
**6d**	*n*-Bu	-	-	4	70	220–222

^a^Isolated yields.

## Conclusion

In conclusion, our results demonstrate a simple, mild and efficient method for the synthesis of novel functionalised pyrimido [4,5-*c*][1,2]diazepines and pyrazolo [3,4-*d*]pyrimidines of biological significance. Heteroannulation on the nucleophilic double bond of uracil usually requires either forcing conditions or relatively long and complex synthetic pathways. Our results have demonstrated that heteroannulation on the double bond of uracil is possible under simple and mild condition using suitable organic substrates.

## Supporting Information

File 1 contains full experimental data

## References

[1] Lunt E, Barton D H R, Ollis W D (1979). Comprehensive Organic Chemistry.

[2] Brown D J, Katritzky A R, Rees C W (1984). Comprehensive Heterocyclic Chemistry.

[3] Sasaki T, Minamoto K, Suzuki T, Yamashita S (1980). Tetrahedron.

[4] Jones A S, Sayers J R, Walker R T, Clercq E D (1988). J Med Chem.

[5] Mitsuya H, Yarchoan R, Broder S (1990). Science.

[6] Pontikis R, Monneret C (1994). Tetrahedron Lett.

[7] Billings B K, Wagner J A, Cook P D, Castle R N (1975). J Heterocycl Chem.

[8] Miyasaka T, Tanaka H, Baba M, Huyakawa H, Walker R T, Balzarini J, Clercq E D (1989). J Med Chem.

[9] Taylor E C, Sawinski F (1974). J Org Chem.

[10] Wamhoff H, Winfried S (1986). J Org Chem.

[11] Hirota K, Benno K, Yumuda Y, Senda S (1985). J Chem Soc, Perkin Trans 1.

[12] Sasaki T, Minamoto T, Suzuki T, Suguira T (1978). J Am Chem Soc.

[13] Blank H U, Fox J J (1968). J Am Chem Soc.

[14] Wexlar A, Swenton J S (1976). J Am Chem Soc.

[15] Hyatt J A, Swenton J S (1972). J Am Chem Soc.

[16] Yoneda F, Higuichi M, Nagamatsu T (1974). J Am Chem Soc.

[17] Yoneda F, Nagamatsu T, Senga K (1977). J Chem Soc, Perkin Trans 1.

[18] Maki Y, Tauta K, Suzuki K (1971). J Chem Soc, Chem Commun.

[19] Sutcliffe E Y, Zee-Cheng K Y, Cheng C C, Robins R K (1962). J Med Chem.

[20] Elion G B, Callahan S W, Nathan H, Bieber S, Rundles R W, Hilching G H (1963). Biochem Pharmacol.

[21] Elion G B, Hand B (1978). Exp Pharmacol.

[22] Prajapati D, Thakur A J (2005). Tetrahedron Lett.

[23] Gohain M, Prajapati D, Gogoi B J, Sandhu J S (2004). Synlett.

[24] Gohain M, Prajapati D, Sandhu J S (2004). Synlett.

[25] Mallory W R, Morrison R W, Styles V L (1982). J Org Chem.

[26] Morrison R W, Styles V L (1982). J Org Chem.

[27] Styles V L, Morrison R W (1982). J Org Chem.

[28] Sternbach L H, Garathini S, Mussini E, Randall L O (1973). The benzodiazepines.

[29] Sharp J T, Katritzky A R, Rees C W (1984). Comprehensive Heterocyclic Chemistry.

[30] Marc G, Pecar S (1998). Synth Commun.

[31] Liu J-F, Kaselj M, Isome Y, Chapnick J, Zhang B, Bi G, Yohannes D, Yu L, Baldino C M (2005). J Org Chem.

[32] Bohlmann F, Rahtz D (1957). Chem Ber.

[33] Ciciani G, Piaz V D, Chimichi S (1984). Heterocycles.

[34] Ege S N, Carter M L C, Spencer R L, Nordman C E, Friedman H Z (1976). J Chem Soc, Perkin Trans 1.

