# Supplementary material for: One-pot synthesis of novel 1H-pyrimido[4,5-c][1,2]diazepines and pyrazolo[3,4-d]pyrimidines
Source: Beilstein J Org Chem. 2006 Mar 23;2:5. doi: 10.1186/1860-5397-2-5 (PMC1470455; doi:10.1186/1860-5397-2-5)
Supplement: File 1 — contains full experimental data [file Beilstein_J_Org_Chem-02-05-s001.zip › Experimental Section.doc]

Experimental Section

General: All reagents are commercially available and were used without further purification. Solvents were dried by standard methods. Melting points were determined by using a Buchi melting point apparatus and are uncorrected. IR spectra were recorded for KBr discs on a Perkin-Elmer 240C analyser. The 300 MHz NMR spectra were recorded on Bruker AC 300 instrument with tetramethylsilane as internal standard. Mass spectra were recorded in an AEIMS-30 spectrometer. Elemental analyses were performed on a Hitachi 026 CHN analyser. The progress of most reactions was monitored by TLC and chromatographic purification was performed with silica gel 60 (120 mesh, Merck).

**Reaction of 1,3-dimethyl-6-hydrazinouracil 1 with enones 2 and α−ketoalkynes 8 : General procedure**: In a very simple experimental procedure, equimolar quantities of 1,3-dimethyl-6-hydrazinouracil **1** (0.17g, 1 mmol) and α,β−unsaturated carbonyl compounds **2a** (0.24 g, 1 mmol) were refluxed in dry ethanol (10 ml) for 3 h, the progress of the reaction was monitored by thin layer chromatography. The suspension of 1,3-dimethyl-6-hydrazinouracil first disappeared with the addition of α,β−unsaturated carbonyl compounds to a clear solution. After completion on usual work up, the product pyrimido[4,5-*c*][1,2]diazepine-6,8-dione **3a** was obtained in 85% yield and this was further recrystallized from ethanol to get the pure product. Similarly other α,β−unsaturated carbonyl compounds **2b-e**, mesityl oxide and alkynones **8** were reacted with 1,3-dimethyl-6-hydrazinouracil **1** and the corresponding pyrimidodiazepines **3** or **4** and pyrazolo[3,4-d]pyrimidines **6** were isolated in 78-90% yields (Table). The characteristics of these compounds are recorded below.

**3a**. mp. 198-200 ^o^C, ^1^H NMR 300 MHz (CDCl_3_) δ 3.31 (s, 3H), 3.67 (s, 2x3H), 3.19 (dd, *J* = 13.7, 2.2, 1H), 3.39 (dd, *J* = 13.7, 5.8, 1H), 4.83 (dd, *J* = 5.4, 2.0, 1H), 6.69 (d, *J* = 8.6, 2H), 7.07-7.47 (m, 7H), 7.81 (1H, D_2_O exchangeable). ^13^C δ 28.4, 29.4 (2xCH_3_), 38.3 (CH_2_), 40.7 (CH), 55.2 (CH_3_), 93.7, 113.7 (2xCH), 126.3 (2xCH), 128.1 (2xCH), 128.4 (2xCH), 129.3 (CH), 136.4, 137.8 146.8, 151.1, 158.2, 161.6, 162.1. MS *m/z* 390 (M^+^), 287 (M^+^ -PhCN); IR (KBr) 3389 (N-H stretch), 1697, 1607 (imide system), 1522 (N-H bending). CHN analyses (calcd %) C, 67.69, H, 5.6; N, 14.35, (found%) C, 67.76; H, 5.49; N, 14.26.

**3b** : Mp 225-26 ^o^C, ^1^H NMR, 300 MHz (CDCl_3_) δ 3.09 (dd, *J* = 13.5, 2.0, 1H), 3.36 (s, 2x, 3H), 4.89 (m, 2H), 6.09-7.02 (m, 10H), 7.76 (1H, D_2_O exchangeable). ^13^C NMR δc 28.4, 29.4 (2 x CH_3_), 31.2 (CH), 38.1 (CH_2_), 86.3, 128.6 (2 x CH), 128.4 (2 x CH), 129.0 (2 x CH), 127.9 (2 x CH), 130.8 (CH), 125.7 (CH), 140.2, 155.0, 155.6, 162, 153.5. MS *m/z* 360 (M^+^), IR (KBr), V_max_ 3379 (brs, N-H stretching), 1697, 1702 (imide system), 1521 (NH bending), CHN analyses (calcd.) C, 70.00; H, 5.55; N, 15.56; (found) C, 70.10; H, 5.42; N, 15.62%.

**3c** : Mp 158-160 ^o^C, ^1^H NMR, 300 MHz (CDCl_3_) δ 0.91 (s, 3H), 3.29 (s, 2x, 3H), 2.91 (dd, *J* = 13.3, 2.1, 1H), 5.57 (m, 2H), 7.01-7.38 (m, 5H), 7.73 (1H, D_2_O exchangeable). ^13^C NMR δc 16.2 (CH_2_), 28.4, 29.4 (2 x NCH_3_), 31.7 (CH), 30.1 (CH_2_), 86.3, 128.4 (2 x CH), 125.0 (2 x CH), 127.9, 153.5, 155.5, 155.6, 161.9. MS *m/z* 298 (M^+^), IR (KBr), V_max_ 3387 (N-H stretching), 1716, 1696 (imide system), 1521 (NH bending), CHN analyses (calcd.) C, 64.43; H, 6.04; N, 18.68; (found) C, 64.32; H, 6.10; N, 18.75%.

**3d** : Mp 192-93 ^o^C, ^1^H NMR, 300 MHz (CDCl_3_) δ 1.01 (s, 1H), 3.02 (dd, *J* = 13.6, 2.2, 1H), 3.28 (dd, *J* = 13.6, 5.6, 1H), 3.56 (s, 2x, 3H), 3.96 (dd, *J* = 5.6, 2.0, 1H), 6.65 (d, *J* = 8.6, 2H), 6.95 (d, *J* = 8.6, 1H), 7.72 (s, 1H, D_2_O exchangeable). ^13^C NMR δ 28.4, 29.4 (2 x NCH_3_), 38.2 (CH_2_), 86.1, 128.4 (2 x CH), 127.0 (2 x CH), 129.7, 155.6, 155.0, 186.3, 161.9, 153.5, 151.1. MS *m/z* 332 (M+), IR (KBr), V_max_ 3386 (N-H stretching), 1696, 1712 (imide system), 1525 (NH bending), CHN analyses (calcd.) C, 57.74; H, 5.11; N, 16.84; (found) C, 57.65; H, 5.01; N, 16.89%.

**3e** : Mp 190-91 ^o^C, ^1^H NMR, 300 MHz (CDCl_3_) δ 0.08 (s, 3H), 3.28 (s, 2x, 3H), 4.84 (m, 2H), 5.56 (m, 2H), 7.81 (s, 1H, D_2_O exchangeable). ^13^C NMR δc 15.7 (CH_3_), 28.4, 29.4 (2 x NCH_3_), 56 (CH_2_), 86.4, 129.4, 155.5, 153.5, 162.9. MS *m/z* 222 (M^+^), IR (KBr), V_max_ 3386 (N-H stretching), 1676, 1710 (imide system), 1520 (NH bending), CHN analyses (calcd.) C, 54.55; H, 6.36; N, 25.45; (found) C, 54.45; H, 6.25; N, 25.50%.

**4**: Mp 156-57 ^o^C, ^1^H NMR 300 MHz (CDCl_3_) δ 0.94 (s, 3H), 1.41 (s, 3H), 1.86 (d, *J* = 12.7, 1H), 2.25 (s, 3H, overlapping possibly with 1H, d of *J* = 12.7 at ~δ 2.83), 3.27 (s, 3H), 3.32 (s, 1H), 3.48 (s, 3H) with no D_2_O exchangeable signal. ^13^C δc 24.7, 25.8, 27.4, 28.0, 29.9 (5xCH_3_), 44.4 (CH_2_), 49.2 (CH), 53.5, 147.5, 151.1, 162.3, 166.6. MS m/z 250 (M^+^), 235 (M^+^ -15); IR V_max_ 1718, 1676 (imide system). 1589 (C=N) cm^-1^, no NH/OH absorption. CHN analyses (calcd %) C, 57.60; H, 7.20; N, 22.40; (found %) C, 57.51; H, 7.15; N, 22.46.

**6a, 6c**: mp 237-239 ^o^C. ^1^H NMR 300 MHz (CDCl_3_) δ 1.37 (t, *J* = 7.1, 3H), 1.77 (br, NH), 3.08 (q, *J* = 7.1, 2H), 3.28 (s, 3H), 3.52 (s, 3H); ^13^C δc 24.6, 27.4, 29.1, 41.0, 107.0, 130.1, 157.7, 158.4, 160.3. MS m/z 209 (M+), IR V_max_ 3225, 3210, 1700 cm-1. CHN analyses (calcd %) C, 51.92; H, 5.77; N, 26.92; (found %) C, 52.01; H, 5.82; N, 26.83.

**6b**: Mp 242-244 ^o^C. ^1^H NMR 90 MHz (CDCl_3_) δ 1.85 (br, NH), 3.22 (s, 3H), 3.48 (s, 3H), 7.10-7.43 (m, 5H); ). ^13^C NMR δc 28.6, 29.2, 106.8, 111.7, 115.7, 120.0, 124.6, 129.6, 158.0, 158.4, 160.0. MS m/z 257. (M+), IR (KBr), V_max_ 3250, 3205, 1700, cm-1. CHN analyses (calcd %) C, 65.62; H, 4.68; N, 21.87; (found %) C, 6571; H, 4.75; N, 21.79%.

**6d**: mp 220-222 ^o^C. 1H NMR 90 MHz (CDCl_3_) δ 0.94 (t, *J* = 7.0, 3H), 1.45 (m, 2H), 1.58 (m, 2H), 1.80 (br, NH), 3.15 (t, *J* = 6.7, 2H), 3.68 and 3.48 (s, 6H, 2Me); ^13^C δc 14.0, 22.9, 24.8, 28.3, 30.1, 32.8, 106.3, 128.1, 152.8, 157.6, 161.6. MS m/z 236 (M+), IR V_max_ 3225, 3210, 1700 cm-1. CHN analyses (calcd %) C, 55.93; H, 6.78; N, 23.73; (found %) C, 55.87; H, 6.85; N, 23.66.
